# Reconstructive Surgery of the Head and Neck in Organ Transplant Recipients: A Case Report and a Review of the Literature

**DOI:** 10.3390/jcm13164790

**Published:** 2024-08-14

**Authors:** Andrea Rampi, Lara Valentina Comini, Andrea Galli, Bright Oworae Howardson, Alberto Tettamanti, Paolo Luparello, Gabriele Redaelli, Davide Di Santo, Stefano Bondi

**Affiliations:** 1 Otorhinolaryngology Unit, Sondrio Hospital, ASST Valtellina e Alto Lario, 23100 Sondrio, Italy; 2 Otorhinolaryngology, Head and Neck Surgery, Candiolo Cancer Institute, FPO-IRCCS, 10060 Turin, Italystefano.bondi@ircc.it (S.B.); 3 Otorhinolaryngology Unit, Division Head and Neck Department, IRCCS San Raffaele Scientific Institute, 20132 Milan, Italy; 4 School of Medicine, Vita-Salute San Raffaele University, 20132 Milan, Italy

**Keywords:** head and neck surgery, solid organ recipients, wound healing, immunosuppressive agents, reconstructive flaps

## Abstract

The number of solid organ transplant recipients (SOTRs) is growing as a consequence of an increase in transplantations and longer survival; these patients, thus, frequently suffer various comorbidities and are subjected to the detrimental effects of immunosuppressive agents, which expose them to a higher risk of developing malignancies. These drugs also complicate the surgical treatment of neoplasms, as they can hinder wound healing, especially when associated with other unfavorable factors (e.g., previous radiotherapy, diabetes, etc.). We herein present our experience with a 74-year-old SOTR who underwent a radical extended parotidectomy and reconstruction with a submental island flap for a persistent cutaneous squamous carcinoma after radiotherapy; his complicated clinical course was characterized by incredibly slow wound healing. The current literature was reviewed to provide a succinct overview of the main difficulties of head and neck surgery in SOTRs. In particular, the immunosuppressive regimen can be tapered considering the individual risk and other elements should be carefully assessed, possibly prior to surgery, to prevent cumulative harm. New developments, including intraoperative monitoring of flap vascularization through indocyanine green fluorescence video-angiography and the prophylactic application of negative pressure wound therapy, when feasible, may be particularly beneficial for high-risk patients.

## 1. Introduction

Solid organ transplant procedures have increased in recent years, leading to a growing cohort of long-survivor patients receiving chronic therapy with immunosuppressive agents, which are known to have high potential impacts in the oncologic field [1,2,3,4]. In particular, solid organ transplant recipients (SOTRs) have been shown to develop malignant neoplasms with a frequency that is up to five times higher than the general population and which increases proportionally with the time from transplant [1,3]; the ear-nose-throat (ENT) district is not devoid of these implications, resulting, in particular, in a substantial increase in the incidence of skin tumors, squamous cell carcinoma of the upper airways, and sarcomas [1,2,3,5].

Pharmacological therapies to prevent organ rejection are not only risk factors for the development of malignancies but may also represent a major obstacle for surgical treatment; indeed, they have an antiproliferative activity on endothelial cells, fibroblasts, and smooth muscle cells, consequently hindering inflammatory and reconstructive processes that are necessary for proper healing [6,7].

The presence of other conditions negatively impacting wound healing, such as previous radiotherapy (RT) diabetes mellitus, malnutrition, advanced age, smoking habit, alcohol consumption, and endocrinological disorders increases post-surgical risks [8,9,10]; ENT surgeons having to deal with wound healing in these extremely difficult cases often suffer frustrating failures, and the literature is scarce on the management of such specific and difficult-to-treat patients [5].

We herein present our experience with a 74-year-old liver transplant recipient, who underwent salvage surgery following radiotherapy for a persistent skin squamous cell carcinoma (SCC) with parotid involvement; the pedicled submental island flap (SIF) used for the reconstruction of the surgical deficit suffered severe difficulties in the engraftment process and required prolonged wound care. In addition, the literature was reviewed in search of other cases of reconstructive salvage surgery in transplant recipients and how postsurgical complications were managed; papers in PubMed, Scopus, and Cochrane databases were also searched for the most updated data on the epidemiology and etiology of cancers (in particular head and neck malignancies) in SOTRs, as well as recommendations regarding surgery, tapering of immunosuppressive therapy during the perioperative period and management of complications in this population.

## 2. Case Presentation

A 74-year-old man was referred to our department for a persistent SCC of the skin of the auricle following upfront RT performed at another hospital. The patient had a history of liver transplantation for alcoholic cirrhosis 4 years prior and since then he had been on an immunosuppressant regimen consisting of 3 mg of tacrolimus (TR) daily and 500 mg of mycophenolate mofetil (MMF) twice daily. His complex medical history also included hypersplenism-induced leukopenia and thrombocytopenia, portal hypertension causing esophageal varices and gastropathy, and previous infection with HAV and HBV. The RT regimen consisted of a total RT dose of 5250 cGy in 10 fractions of 525 cGy each, administered between December 2021 and February 2022. Unfortunately, no further information was available regarding the staging of the disease prior to RT.

**Figure 1:**
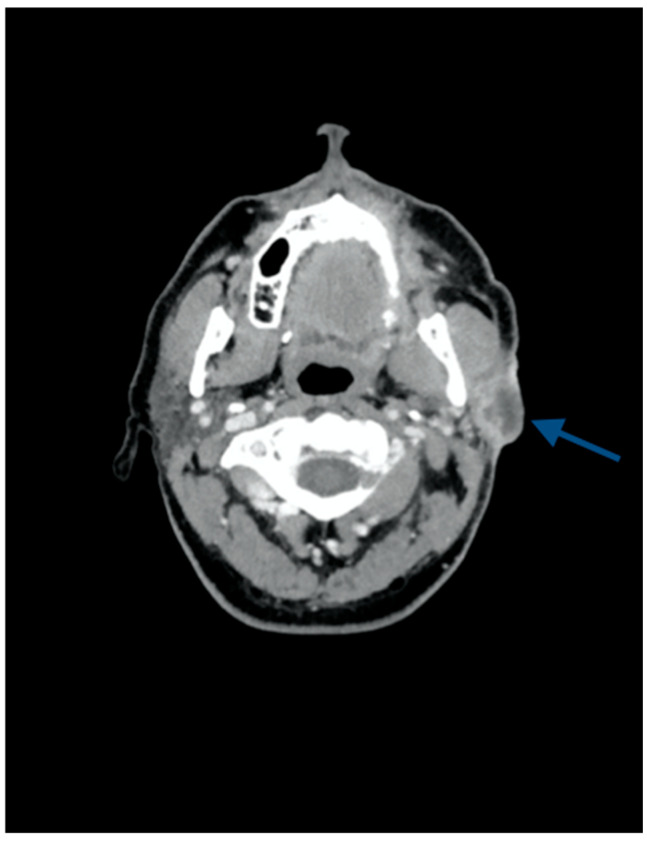
A CT scan displaying the skin neoplasm with deep invasion of the underlying parotid gland, the masseter muscle, and the anterior wall of the external auditory canal in its cartilaginous portion.

Two months after the end of primary RT, the patient developed an ulcerated lesion of the left preauricular region causing an antalgic trismus and a House–Brackmann grade IV facial paralysis. A CT scan confirmed the presence of a 2.9 × 2.2 × 5.5 mm skin neoplasm with deep invasion of the underlying parotid gland, the masseter muscle, and anterior wall of the external auditory canal in its cartilaginous portion; no obvious bone infiltration or lymph node metastases were detected (Figure 1).

In our department, he therefore underwent, in June 2022, a radical extended parotidectomy with exposure of the mandibular condyle, concurrent selective neck dissection (levels IB-II-III-IV), and reconstruction with a pedicled SIF. The pathology report confirmed a persistent skin SCC, staged (y)(r)pT3pN0 (0/24) L1 Pn1 according to the TNM, VIII edition, with clear margins (R0).

The perioperative management of his immunosuppressant therapy was previously discussed with the transplant team: MMF was suspended, while dose reduction of TR dosage was not advocated, even though blood levels were tested every 3 days after surgery and the dosage was adjusted to maintain its levels between 5 to 8 ng/mL, according to current recommendations [11,12].

**Figure 2:**
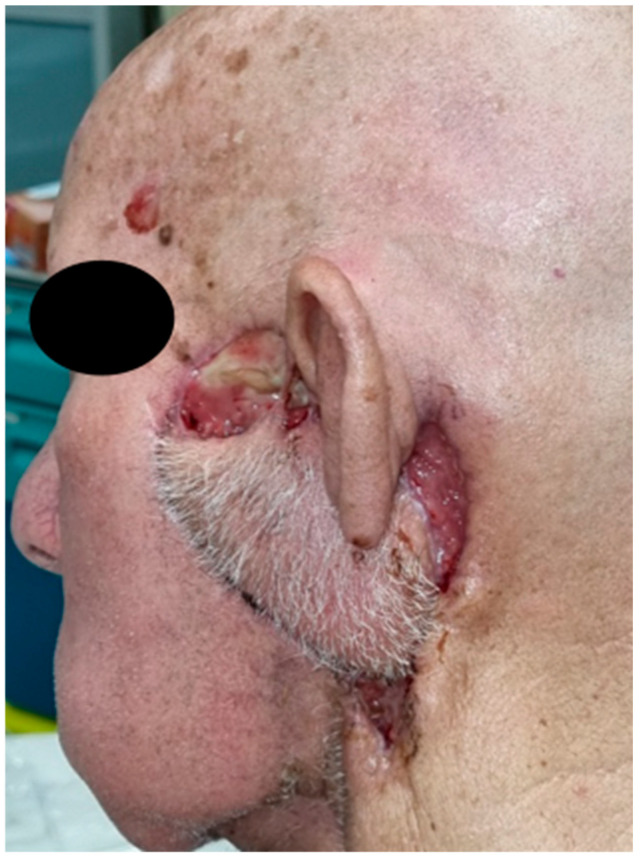
Multiple wound dehiscences in the pre-, infra- and retro-auricular region, with exposure of the mandibular condyle and the mastoid. No evidence of flap failure, which appears to be trophic and well-vascularized.

Three days after surgery, the patient presented a fever (>38 °C), inappetence and significant asthenia; physical examination revealed a centimetric retroauricular dehiscence of the surgical wound with purulent discharge; no obvious flap necrosis was observed. Blood tests showed increased inflammatory markers and microbiological tests on wound discharge were positive for *Pseudomonas aeruginosa*, *Enterobacter aerogenes*, and *Klebsiella oxytoca*; therefore, antibiotic therapy was switched from a first-generation cephalosporin (Cefazolin 1 g, which was administered as regular 48 h post-operative antibiotic prophylaxis) to piperacillin/tazobactam 4 g/0.5 g four times per day. Despite the improvement in blood tests and the disappearance of fever, a progressive worsening of wound dehiscence was observed: eight days after surgery, the patient presented with multiple wound dehiscences with exposure of the mandibular condyle and the mastoid, although without evidence of flap failure (Figure 2).

**Figure 3:**
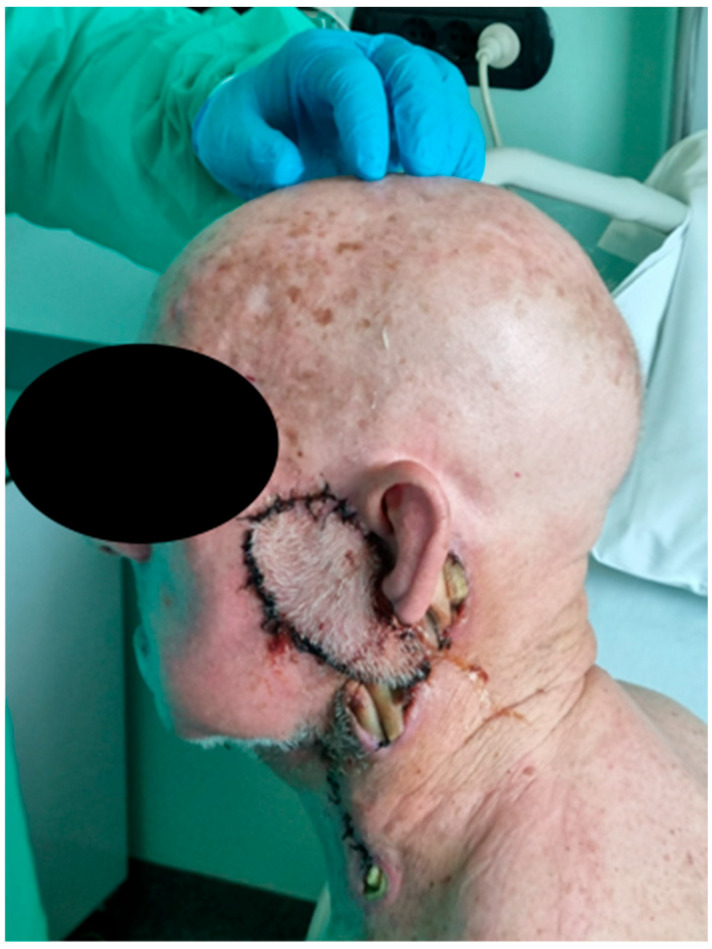
Advanced wound care.

Wound care specialists were consulted: wound irrigation with polyhexanide/betaine-based solution (Prontosan^®^ Wound Irrigation Solution) was suggested for proper cleansing, and dressings with Ag-based hydrofiber and hydrophobic polyurethane foam (Askina^®^ Calgitrol^®^ Ag) were chosen due to the evident infection and significant discharge from the surgical site (Figure 3).

The patient’s white blood cells (WBC) had been roughly stable during initial immunosuppressive therapy and following RT, ranging from 3.0 to 3.5 × 10^9^/L, reaching between 4.0 and 4.5 during the infection’s peak, and slowly returning to previous levels. Analogously, the patient’s platelet levels ranged from 60 to 70 × 10^9^/L, and a reduction (from 50 to 55 × 10^9^/L) was observed during the infection. Leukopenia and thrombocytopenia were likely multifactorial, with hypersplenism and liver cirrhosis playing a primary role; previous RT and immunosuppressive agents, despite a more limited impact on WBC, were still considered to significantly impact immunological response and healing.

The surgical wound showed a progressive, but incredibly slow, improvement: the patient was discharged after 14 days of systemic antibiotic therapy with no signs of residual infection, but only a modest reduction in the wound dehiscence; a renewal of the dressing by home nursing carers every 48–72 h was necessary over a period of approximately 6 months to allow a complete closure of the defect. No growth factors were administered to prevent eventual stimulation of residual neoplastic cells, and negative pressure wound therapy (NPWT) was excluded due to practical difficulties regarding its long-term application on an outpatient basis. As of today, regular clinical and radiological follow-up has shown no recurrence of disease.

## 3. Discussion

In recent times, many fields in medicine and surgery have been challenged by the consequences of their own success: the increasing efficacy of different therapies has increased the prevalence of patients suffering from various comorbidities and receiving multiple therapeutic agents, with an obvious impact on the difficulty of their treatment [2,3]. In the field of organ transplantation, continuous technical and pharmacological advances have led to a substantial increase in the survival of SOTRs, who are nonetheless subjected to the negative consequences of chronic immunosuppression [1,3,6,7,13,14]. Of particular interest to the ENT surgeon are the increases in the incidence and aggressiveness of head and neck malignancies, along with the significant delay in wound healing [15,16].

Indeed, frustratingly slow tissue renewal may be experienced in patients receiving immunosuppressant therapy for an organ transplant, especially if it is added to other detrimental factors, as in the case of our patient (a 74-year-old, previously irradiated and suffering from leuko- and thrombocytopenia).

The literature has, in fact, widely discussed the significant impact of RT on the process of wound healing; the roles of obesity, diabetes mellitus, malnutrition, sarcopenia, advanced age, smoking, alcohol consumption and endocrinological disorders have also been extensively assessed, with a risk of impaired healing that exponentially increases when these conditions are combined [8,9,17,18].

The literature on head and neck reconstructive surgery in SOTRs is, unfortunately, extremely limited. Miller et al. [19], in 2011, presented a worrisome report of 11 complications (45% major) on 22 free flaps in 8 of 17 SOTRs, noting that 36% of patients and 45% of complicated flaps had a history of previous irradiation.

Sbitany et al. [20] reported on twenty-four free tissue transfers in SOTRs, of which only eight were for the reconstruction of the head and neck region; among these, two suffered from delayed healing. Interestingly, the authors reported a significant association with the use of prednisone as the immunosuppressive drug and perioperative morbidity; sirolimus was not related to delayed healing, although it was administered in three of four patients suffering from vascular thrombosis. Particularly interesting is the case series by the MD Anderson Cancer Center [21], with 25 SOTRs receiving 28 free tissue transfer flaps (in 5 cases with a history of previous RT): despite particularly accurate perioperative management of patients, surgical complications affected 13 cases, although mainly mild sequelae were reported (primarily hematoma or delayed wound healing).

In such a complex scenario, it is far beyond the goals of the present paper to provide guidelines on the management of immunosuppressive therapy in this specific field, which is a prerogative of dedicated experts, but we aim to offer ENT surgeons a succinct overview of the burden and difficulties associated with surgery and wound healing in SOTRs, especially when associated with RT or other hindering factors.

### 3.1. Epidemiology and Etiology of Cancers in SOTRs

SOTRs are, by definition, vulnerable patients: indeed, they receive chronic immunosuppressive agents, exposing them to infections, and generally suffer from a range of comorbidities, that may be unrelated to the cause of the transplant, represent adverse sequelae of the transplant process, or be caused by the same detrimental factors that determined the organ failure (e.g., alcohol consumption in liver recipients for alcoholic cirrhosis) [3,14]. Despite this, cancer is the second leading cause of death in SOTRs, accounting for 13–20% of annual treatment mortalities, with a two- to five-fold increase in the risk of neoplasms compared with the general population [5,14,15,22,23,24,25].

Skin tumors are the most common cancers in SOTRs, characterized by an inversion in the incidence of basal and squamous cell carcinoma, with the latter being far more frequent [3,15]; some authors have suggested considering cutaneous squamous cell carcinoma (cSCC) in SOTRs as a separate entity from cSCC in non-SOTRs given its peculiar aggressiveness and therapeutic needs [25]. Lymphoproliferative malignancies are ranked second in terms of frequency.

The head and neck region, which is involved in less than 4% of cancers in the general population, represents the primary site of approximately 15% of malignancies in SOTRs [15]. In this site, cSCC still represents the majority of cases, whereas mucosal SCC of the upper aerodigestive system has a less pronounced, although still significant, increase in the relative risk (1.67 in a recent wide study, with thyroid and salivary gland tumors reaching 1.85 and 2.91, respectively) [1].

The mechanism underlying the excess of risk in this subpopulation is likely multifactorial: on one hand, tumor-associated factors may also determine organ failure (e.g., sclerosing cholangitis, which increases both liver failure and neoplasia) and end-organ disease may lead to an increase in oncogenic substances; on the other hand, immunosuppressant agents may induce neoplastic transformation through direct carcinogenesis or the indirect effects of immunosuppression [14]. In the latter case, these drugs reduce the possibility of detecting cells with early neoplastic mutations by acting on different immunosurveillance pathways [14]; in addition, the reduced immune response allows easier replication of viruses (such as HPV and EBV), whose ability to induce neoplastic transformation is well known. This effect also appears to be dose-dependent by direct comparison [26,27] and by the apparently higher rate of malignancies in patients receiving an organ transplant with a greater need for immunosuppression (e.g., lung and heart) [14].

### 3.2. Surgical Approach

Specific recommendations for head and neck reconstructive surgery in SOTRs are lacking, mainly due to the rarity of the cases reported. An analogy with patients with previous RT (i.e., salvage surgery), whose role has been extensively studied, is thus advisable [28]. Radiation has, in fact, a consistent effect on wound healing, provoking vascular damage, tissue hypocellularity, and fibrosis, which makes the surgical field less able to counteract any detrimental factors [28].

The specific role of chemotherapy in the damage of local tissues is not equally studied in the literature, especially in the head and neck region, where it is almost invariably associated with RT; it is thus rational that the same sensitizing properties to radiation exploited for oncologic purposes may apply to the actinic damage to tissues. Chemotherapy agents also have a systemic impact that may act synergically to impair wound healing [26].

In this scenario, the use of a vascularized flap to provide additional healthy tissue to the surgical field has been shown to reduce post-surgical complications in the head and neck region (e.g., fistulas after laryngectomy) and the transfer of muscular, highly vascularized tissue has been linked to better outcomes [28]. A personalized approach is obviously necessary, but a lower threshold for SOTRs in this direction appears warranted. Locoregional flaps are often preferred in fragile subjects due to their faster harvesting and insetting, and in most cases (as in our report), they are also chosen for their superior color matching in skin reconstruction; however, the donor site of the flap may have been included in the previous radiation field.

Conversely, the literature on the impact of previous RT on the success rate of anastomosis in free flaps is somewhat contradictory, but in most cases, it appears not relevant [18,29]. Free tissue transfers are therefore preferred by many surgeons for their higher efficacy, and in the largest case series in head and neck surgery in SOTRs, the use of free flaps for reconstruction of the surgical defect is widely reported; it is thus advisable to opt for this type of reconstruction in patients who can tolerate it, in selected centers with significant experience [19,20,21,28]. Nevertheless, it is worth stressing that the simultaneous presence of previous irradiation and immunosuppressive therapy, as in our patient, exponentially increases the risk of a poor wound outcome, regardless of the reconstructive strategy adopted.

### 3.3. Immunosuppressant Therapy and Wound Healing

Immunosuppressant agents are the mainstay to avoid rejection in SOTRs, usually distinguished as induction and maintenance therapy, but they are related to an increased risk of developing malignancies and to delayed/insufficient wound healing [3,5,13,30]. The latter process normally follows a well-structured and sequential pathway: at the beginning, tissue damage is followed by hemostasis and the release of inflammatory mediators that are responsible for the chemotaxis of different cells involved in the inflammatory process [9,30]. These cells are responsible for clearing the tissue and producing different chemokines which, in turn, induce a proliferative phase characterized by neoangiogenesis, matrix deposition, and the development of granulation tissue [9,30]. The remodeling phase starts with a partial overlap with the proliferative one: as soon as the extracellular matrix is produced, it starts to be remodeled with subsequent healing of tissue and eventual scar formation [9,30].

Immunosuppressant agents hinder this process by acting on different targets, mainly inhibiting inflammatory and replicative mediators, and are generally recognized as having a significant impact on the corresponding steps in wound healing [13,30]. Among these agents, corticosteroids have long been the mainstay of therapy, but the introduction of more specific and less toxic drugs has led to a reduction in their use; they are currently mostly used in the induction phase or in the treatment of re-exacerbations [31]. MMF is an antimetabolite drug that interrupts purine biosynthesis in T and B lymphocytes [30]. Cyclosporine inhibits calcineurin, which is an important factor for the production of various cytokines (IL-2, IFN-γ, GM-CSF, TNF-α) involved in T-cell differentiation; TR analogously inhibits calcineurin activity with a different mechanism but a power that is 10–100-fold higher than cyclosporine; sirolimus blocks the activity of a mTOR kinase, which is crucial in several cell functions such as cell replication and in the response to IL-2, IL-15, and VEGF [31]. As an example, to understand the numerous variables to be considered in the management of SOTRs, a switch from a calcineurin inhibitor to sirolimus has been shown to decrease the risk of skin cancer, but has also been associated with hypertension, hyperlipidemia, lung toxicity, and impaired wound healing [32]. In particular, its correlation with wound complications has been mainly investigated in transplant surgery, with evidence of a significant increase in postsurgical wound morbidity in renal recipients receiving sirolimus rather than TR, in addition to MMF and prednisone [33,34]. The detrimental effect of sirolimus on wound healing is so consistent that some authors advocate avoiding both sirolimus and everolimus (a derivative with similar features but a shorter half-life) in the perioperative period of transplant surgery to avoid such complications [31].

### 3.4. Wound Healing Management in SOTRs: A Pragmatic Approach

Because the literature is particularly lacking in large case series of head and neck reconstruction in previously irradiated SOTRs or indications for their management, many suggestions may be adapted from studies on other body regions or patients with different features. In particular, precautions that are routinely applied to all reconstructive surgeries acquire even more importance in SOTRs. In this scenario, the broad experience at the MD Anderson Cancer Center has brought about the development of a well-integrated multidisciplinary team which discusses all the possible comorbidities and needs of patients before surgery in all head and neck reconstructive procedures [21]. In particular, the more comorbidities the patient suffers from, the more strictly he/she should be assessed in the perioperative period to reduce any possible synergic detrimental effects, possibly as early as surgery is indicated. Concerning possible delayed wound healing, the patient’s glucose levels should be assessed and aggressively treated as needed, while analogous considerations apply to other endocrinological disorders, especially hypothyroidism. Nutritional deficiencies, particularly in the elderly, should be corrected in advance, with particular attention to sufficient protein intake, which may also have positive effects on skeletal muscle mass depletion and sarcopenia. Perioperative antibiotics should be carefully evaluated, taking into account the propensity of these patients to infections and the apparently lower rate of complications after transplant surgery due to specific antibiotic prophylaxis [35]; in our case, an antibiotic preventive regimen was set, but it ended up being insufficient.

In addition, predominant attention should be paid to immunosuppressive therapy during the entire perioperative period; in particular, a reduction in immunosuppressive therapy is advised by many authors, as performed in a considerable proportion of patients in the previously mentioned experience by Schaverien et al. [21] (and in our case). Cooperation with a transplant expert is therefore essential to determine the risk of rejection based on the organ involved, previous medical history, the age of the patient, or antigen mismatches [21,35].

Analogously, a switch to immunosuppressant agents with less impact on wound healing has to be considered: in fact, based on the present literature avoiding sirolimus and corticosteroids appears to be a valid option, when feasible [31,35]. Frequent monitoring of the transplanted organ is obviously essential in this phase, especially when a change in treatment is made.

Concerning specific indications for head and neck reconstructive techniques in SOTRs, other than the previously mentioned comparison of free and pedicled flaps, the literature is unfortunately lacking; a previous report [35] on wound healing after kidney or liver transplant highlighted the predominant role of intraoperative contamination, prolonged preoperative stay, and hypothermia on wound healing. Despite the limited evidence, it is thus reasonable that normal precautions, such as tension-free reconstruction or the removal of insufficiently vascularized tissue, should be implemented. In this regard, the recent introduction in head and neck surgery of intraoperative monitoring of flap vascularization through indocyanine green (ICG) fluorescence video-angiography has provided the surgeon with a reliable and quantitative tool, allowing for eventual targeted trimming of hypovascularized areas based upon a flap-to-normal skin/mucosa ICG ratio [36,37,38]. The authors have increasing confidence in the use of this technology and advocate its adoption in all cases of head and neck flaps, especially in patients with multiple risk factors for vascular impairment.

Our case aligns with the previously mentioned case series [19,20,21] that report a high percentage of postoperative complications in SOTRs despite several precautions; postoperative care and monitoring are therefore fundamental. Schaverien et al. [21] have advocated a rapid awakening protocol in substitution of overnight ventilation under sedation due to the risk of hypotension and consequent fluid overload. A strict monitoring protocol is also necessary for early detection of any complications and wound dehiscences; in this case, advanced wound care techniques should be immediately introduced after multidisciplinary discussion with wound care specialists (as adopted in our patient). Analogously to the surgical approach, there are no specific indications for wound healing in SOTRs, but the most appropriate strategy should be chosen depending on factors such as the amount of discharge or the presence of obvious infection of the wound.

In light of the high risk of infection, it is worth noting that wound care agents with antimicrobial properties may paradoxically impair the wound healing process, and there is no evidence of the benefit of their prophylactic use in uninfected fields [39]. In the event of a local infection, the choice of local antibiotic creams must be targeted using a specific antibiogram. Analogously, some evidence in the literature, although limited, supports the use of iodopovidone dressings over silver dressings for their inferior cytotoxicity [39]. In recent times, researchers have been investigating the biological mechanisms of bacterial wound impairment, including the competitive or synergic mechanisms between different microorganisms and the crucial role of wound microbiota [10]. This has led to studies not only on new targeted antimicrobial agents, but also on the possible use of skin probiotics [10].

An important tool, which can operate synergically with other techniques, is represented by NPWT: this system, by applying negative pressure on the surgical site previously prepared with proper foams and films, has demonstrated an increase in the rate of healing [40]. Its application to the head and neck region has been slower given the difficulties in its application due to the complex topography and presence of different orifices [40]. It is worth stressing that NPWT must be avoided in the presence of conditions like an active tumor in the wound, untreated osteomyelitis, necrosis, or when in direct contact with a nerve or a vessel, and therefore an expert should be consulted for its application. Nonetheless, a recent review [40] highlighted that the use of this technology is increasing and, with proper adaptations, is now used even in conditions that were previously considered contraindications (e.g., pharyngeal phistulas) [41]. A recent systematic review investigated the results of its immediate application (on day one) at the site of insetting of free flaps for head and neck reconstruction, reporting a successful result in 54 out of 56 flaps [41]. The prophylactic application of NPWT may therefore represent an option for patients with a significant risk of wound healing, such as in SOTRs.

## 4. Conclusions and Future Perspectives

For decades, ENT surgeons have been facing the difficulties of impaired wound healing after complex surgical procedures, but the increasing prevalence of multiple risk factors is raising the bar in this field. In light of this, immunosuppressive therapy in SOTRs represents one of the prevalent conditions to consider, and every attempt should be made to inhibit its detrimental effects, including partial withdrawal of immunosuppressive therapy. In the future, better definition of the specific role of any of these drugs is desirable, as well as personalized care based on the specific alteration of the healing [42]. Moreover, research in wound care techniques is providing better agents that promote specific healing pathways or better delivery of those already known; the recent introduction of growth factors is particularly interesting, whose use to date has been limited to non-oncologic fields to avoid the induction of neoplastic proliferation. The prophylactic use of NPWT appears to be another valuable tool to consider [42].

## Data Availability

No new data were created in this study. All the papers considered for the review are publicly available in PubMed, Embase or Scopus.
